# Supplementary material for: SplicedFamAlign: CDS-to-gene spliced alignment and identification of transcript orthology groups
Source: BMC Bioinformatics. 2019 Mar 29;20(Suppl 3):133. doi: 10.1186/s12859-019-2647-2 (PMC6439985; doi:10.1186/s12859-019-2647-2)
Supplement: Supplementary file 1 — Pseudocode of algorithms for SplicedFamAlign steps. A file at the PDF format describing the algorithms used in the SFA method for the preliminary step of splicing structure prediction (Algorithm 1), the first step of local alignment (Algorithm 2), and the second step of gapped extension of anchors (Algorithm 3). (PDF 110 kb) [file 12859_2019_2647_MOESM1_ESM.pdf]

Additional file 1 – Pseudocode of algorithms for SplicedFamAlign steps

Safa Jammali, Jean-David Aguilar, Esaie Kuitche, Aïda Ouangraoua
Department of Computer Science, Université de Sherbrooke,
Sherbrooke, QC, Canada
Contact: safa.jammali@usherbrooke.ca

Algorithm 1 Splicing structure prediction

INPUT: C: CDS sequence ; G: gene sequence containing exons of C
OUTPUT: *exons*: spliced alignment of C against G
list_of_hits ← list of tblastx hits for C against G (by increasing E-values)
exons ← [ ] ; $i \leftarrow 0$
/*Find a maximum set of compatible hits***/**
while *exons* does not cover all C and $i < length(list\_of\_hits)$ **do**
  $H \leftarrow list\_of\_hits[i]$
  if $PID(H) == 100$ **then**
    H ← extend H in both directions as far as $PID = 100$
  else
    H ← trim H minimally to have a PID of 100
  end if
  if H is compatible with all hits in *exons* **then**
    exons ← add H to *exons*
    refine hit boundaries to maximise canonical splicing sites
  end if
  $i \leftarrow i + 1$
end while
/*Find additional exons***/**
if *exons* does not cover all C **then**
  for $H \in exons$ **do**
    H ← extend H with gaps in G to find neighboring exons
  end for
end if
/*Cover remaining CDS exons using DP algorithm***/**
if *exons* does not cover all C **then**
  exons ← local DP alignments to align remaining segments of C
end if

Algorithm 2 Local alignment

INPUT: $list\_of\_hits$: list of tblastx hits
OUTPUT: $list\_of\_anchors$: list of local alignments
$list\_of\_anchors \leftarrow [\ ]$
/*Step i)***/**
for $H \in list\_of\_hits$ **do**
  $E \leftarrow$ find the exon of the CDS that the hit H covers the more
  $H \leftarrow$ trim the hit H so that H covers only the exon E
  $hits\_of\_exon[E] \leftarrow$ add the hit H to $hits\_of\_exon[E]$
end for
/*Step ii)***/**
for $E \in CDS\_exons$ **do**
  $kept\_hits\_of\_exon[E] \leftarrow [\ ]$
  $hits\_of\_exon[E] \leftarrow$ sort $hits\_of\_exon[E]$ by increasing E-value
  for $H \in hits\_of\_exon[E]$ **do**
    if H is compatible with all hits in $kept\_hits\_of\_exons[E]$ **then**
      $kept\_hits\_of\_exon[E] \leftarrow$ add H to $kept\_hits\_of\_exon[E]$
    end if
  end for
end for
/*Step iii)***/**
for $(E_1, E_2) \in CDS\_exons^2$ **do**
  for $(H_1, H_2) \in kept\_hits\_of\_exon[E_1] \times kept\_hits\_of\_exon[E_2]$ **do**
    if $H1$ and $H2$ are not compatible **then**
      keep the hit with the lower E-value and discard the other
    end if
  end for
end for
/*Step iv)***/**
for $E \in CDS\_exons$ **do**
  $S \leftarrow$ merge $hits\_of\_exon[E]$ into a set S of non-overlapping local alignments (k, l, a, b) of CDS segments (k, l) and gene segments (a, b)
  $list\_of\_anchors \leftarrow$ add set S of local alignments to $list\_of\_anchors$
end for

Algorithm 3 Gapped extension of anchors

```
INPUT: list_of_anchors: list of local alignments
OUTPUT: list_of_ext_anchors: list of extended local alignments
list_of_ext_anchors ← [ ]
for (k, l, a, b) ∈ list_of_anchors do
  (k', l') ← CDS exon covered by (k, l, a, b)
  /***Step i) Extension on the left***/
  if k' < k then
    /***Extension configuration (a)***/
    max_identity_extension ← (k', k, a − (k − k'), a)
    max_identity ← PID(max_identity_extension)
    /***Extension configuration (b)***/
    for i ∈ [1, α/3] do
      extension ← (k', k, a − (k − k') − 3i, a − 3i)
      identity_extension ← PID(extension)
      if identity_extension > max_identity then
        max_identity_extension ← extension
        max_identity ← identity_extension
      end if
    end for
    /***Extension configuration (c)***/
    for i ∈ [1, α/3] do
      extension ← (k', k − 3i, a − (k − 3i − k'), a)
      identity_extension ← PID(extension)
      if identity_extension > max_identity then
        max_identity_extension ← extension
        max_identity ← identity_extension
      end if
    end for
    if max_identity ≥ β then
      left_extension ← extension
    end if
  end if
  /***Step ii) Extension on the right***/
  if l < l' then
    right_extension ← procedure symmetric to the one used in Step i)
    for extension on the left
  end if
end for
(k, l, a, b) ← merge left_extension, (k, l, a, b) and right_extension into a
single extended alignment
list_of_ext_anchors ← add (k, l, a, b) in list_of_ext_anchors
```
